# Comparison of the efficacy of letrozole-ovulation induction cycle, natural cycle, and hormonal replacement therapy cycle in cryopreserved embryo transfer: a retrospective cohort study

**DOI:** 10.3389/fendo.2026.1885248

**Published:** 2026-07-29

**Authors:** Keying Pan, Wenhan Ju, Yue Wang, Qianwen Zhang, Ruyi Wang, Xingyue Jiang, Hongyan Zhang, Shuangbin Shi, Shuai Zhao

**Affiliations:** 1 First Clinical Medical School, Shandong University of Traditional Chinese Medicine, Jinan, China; 2 Guanghua Clinical Medical School, Shanghai University of Traditional Chinese Medicine, Shanghai, China; 3 School of Traditional Chinese Medicine, Shandong University of Traditional Chinese Medicine, Jinan, China

**Keywords:** frozen embryo transfer, hormonal replacement therapy cycle, letrozole ovulation induction cycles, natural cycle, retrospective cohort study

## Abstract

**Objective:**

To compare the clinical efficacy of letrozole-induced ovulation cycles, natural cycles, and Hormonal Replacement Therapy cycle in frozen embryo transfer (FET), and to investigate the effects of different endometrial preparation protocols on pregnancy outcomes, hormonal levels, and endometrial receptivity.

**Methods:**

This study was a single-center retrospective cohort study that enrolled a total of 3,068 FET patients who visited our center between January 1, 2023, and December 30,2025. Patients were divided into three groups based on the endometrial preparation protocol: Group A (letrozole ovulation cycle, n=417), Group B (natural cycle, n=887), and Group C (Hormonal Replacement Therapy cycle, n=1,764). The primary outcome measures were clinical pregnancy rate and live birth rate. Secondary outcome measures included early miscarriage rate, ectopic pregnancy rate, serum estradiol and progesterone levels, endometrial thickness, endometrial morphology, and the incidence of endometrial compression. The impact of different protocols on pregnancy outcomes was evaluated using univariate analysis and multivariate logistic regression analysis.

**Results:**

There were no statistically significant differences in baseline characteristics (age, BMI, type of infertility, AMH, etc.) among the three groups (P>0.05). The clinical pregnancy rate (52.5%) and live birth rate (45.3%) in Group A were higher than those in Group C (48.6% and 40.9%, respectively), but the differences were not statistically significant (P>0.05), and were comparable to those in Group B (50.8% and 43.2%, respectively; P>0.05). The early miscarriage rates in Groups A and B (10.5% and 10.4%, respectively) were lower than that in Group C (11.2%), but the difference was not statistically significant (P>0.05). In terms of hormone levels, the E2 levels on the endometrial transformation day were significantly lower in Groups A and B compared to Group C (P<0.001), while the P levels were significantly higher (P<0.001). The proportion of type A endometrium in Groups A and B (75.5% and 77.1%, respectively) was significantly higher than that in Group C (72.1%; P<0.05). The incidence of endometrial cysts (EC) in Groups A and B (35.0% and 34.0%, respectively) was significantly higher than that in Group C (28.6%; P<0.001). Multivariate logistic regression analysis revealed that, compared with Group C, Groups A (aOR=1.254,95% CI 1.032–1.525, P = 0.023) and B (aOR=1.312,95% CI 1.045–1.648, P = 0.019) were independent positive predictors of clinical pregnancy.

**Conclusion:**

Both letrozole ovulation induction cycles and natural cycles achieved similar clinical pregnancy rates and live birth rates in FET, with both values being numerically superior to those observed in hormone replacement therapy cycles, but non-significant. These two protocols may improve pregnancy outcomes by providing a hormonal environment closer to physiological conditions and enhanced endometrial receptivity. For patients with ovulatory function, letrozole ovulation induction cycles and natural cycles are the preferred endometrial preparation regimens for FET.

## 1 Introduction

*In vitro* fertilization-embryo transfer (IVF-ET) technology has made significant progress over the past four decades (1). However, embryo implantation rate remains a critical bottleneck constraining the success of assisted reproductive technologies. In addition to embryo quality, endometrial receptivity is another pivotal factor determining pregnancy outcomes (2, 3). With the widespread adoption of vitrification freezing technology, the number of cycles required for frozen embryo transfer (FET) has surpassed that of fresh cycle transfers, establishing FET as the predominant approach in assisted reproductive technology (4). The success of FET is highly dependent on the synchronous development of the endometrium and the embryo, meaning that the endometrium must possess the capability to accept embryo implantation within a specific “implantation window” (5). Therefore, determining the optimal endometrial preparation protocol to create the most favorable microenvironment for embryo implantation remains a focal point of ongoing research in the field of reproductive medicine.

Currently, the commonly used FET endometrial preparation protocols in clinical practice primarily fall into three categories: Natural Cycle (NC): Suitable for patients with normal ovulatory function. This approach monitors natural follicular development and ovulation, utilizing the patient’s endogenous hormones for endometrial preparation and transformation, followed by timely embryo transfer after ovulation. This protocol closely resembles physiological conditions, requiring minimal or no exogenous hormonal support, but necessitates frequent monitoring with a relatively high cycle cancellation rate (6). Hormone Replacement Therapy (HRT): Also known as the artificial cycle, this approach utilizes exogenous estrogen (e.g., Triptorelin, which is estradiol valerate) to promote endometrial growth. Once the endometrial thickness meets the target criteria, exogenous progestin is administered to induce endometrial transformation. This regimen offers high controllability and flexible scheduling of embryo transfer dates, making it suitable for patients with ovulatory disorders, poor endometrial response, or irregular cycles (7). However, complete reliance on exogenous hormones may lead to excessive estrogen exposure, potentially adversely affecting endometrial receptivity (8, 9). Ovulation Induction Cycle (OIC): The use of drugs such as letrozole to induce single follicle development, mimicking the hormonal changes of a natural cycle. Letrozole, as a third-generation aromatase inhibitor, reduces estrogen levels in the body, thereby relieving negative feedback on the hypothalamus-pituitary axis, promoting endogenous gonadotropin secretion, and inducing follicle development (10). As a third-generation aromatase inhibitor, letrozole reduces endogenous estrogen levels, thereby removes the negative feedback on the hypothalamus-pituitary axis, promotes the secretion of endogenous gonadotropins, and induces follicular development (11, 12).

Regarding the comparative evaluation of the advantages and disadvantages of these three endometrial preparation protocols, existing research conclusions are inconsistent. Some studies indicate that pregnancy outcomes with letrozole cycles and natural cycles are superior to those with HRT cycles (13–15), However, some studies suggest that the three treatment regimens exhibit comparable efficacy (16, 17). These inconsistencies may be attributed to differences in study design, sample size, patient population, and protocol details. Additionally, previous studies have rarely simultaneously compared all three protocols and systematically analyzed their comprehensive effects on hormone levels, endometrial parameters, and receptivity indicators. Therefore, this study aims to systematically compare the clinical efficacy of OIC, NC, and HRT in FET—a single-center, large-sample retrospective cohort study—covering pregnancy outcomes, hormone levels, endometrial thickness and morphology, and endometrial compaction incidence, thereby providing evidence-based guidance for selecting the optimal endometrial preparation regimen in clinical practice.

## 2 Materials and methods

### 2.1 Study design and patient population

This study is a single-center retrospective cohort study approved by the Reproductive Medicine Ethics Committee of Shandong University of Traditional Chinese Medicine Affiliated Hospital (Approval No.SZ2025100227, Approval date: October 2, 2025), with all participants having signed informed consent forms. Clinical data of FET patients who visited our hospital’s Reproductive Center between January 1, 2023, and December 30, 2025, were collected.

#### 2.1.1 Inclusion criteria

1. Age between 20 and 45 years;
2. Undergoing IVF/ICSI for infertility due to tubal factors, male factors, or unknown causes;
3. Performing FET for the first time or for a subsequent cycle;
4. Transplanting frozen embryos at the blastocyst stage (D5/D6) or blastula stage (D3);
5. Complete clinical records available.

#### 2.1.2 Exclusion criteria

1. Concurrent presence of other severe systemic diseases (e.g., Cushing’s syndrome, pituitary tumors);
2. Chromosomal abnormalities in either partner;
3. Moderate to severe endometriosis (AFS stage III-IV);
4. Congenital uterine malformations or untreated uterine cavity lesions;
5. Use of oocyte or sperm donation cycles;
6. Use of preimplantation genetic testing (PGT) cycles;
7. Use of other ovulation-inducing drugs (e.g., clomiphene, gonadotropins) for endometrial preparation cycles;
8. Incomplete clinical data or lost-to-follow-up cases.

### 2.2 Group

According to the inclusion and exclusion criteria, a total of 3,068 FET cycles were included in the analysis. We select different endometrial preparation protocols based on the patient’s individual circumstances: 1.Letrozole-Induced Ovulation Cycle: Suitable for patients with irregular menstrual cycles or ovulatory disorders; 2. Natural Cycle: Preferred for patients with regular menstrual cycles (25–35 days), a clear history of ovulation, and endometrial thickness that meets requirements under natural conditions; 3. Hormonal Replacement Therapy Cycle: Primarily indicated for patients with historically thin endometrium during natural cycles, or those with concurrent adenomyosis, recurrent implantation failure, or severe endometriosis. The patients were divided into three groups based on the endometrial preparation protocol: Group A (letrozole-induced ovulation cycle, n=417): ovulation was induced with letrozole; Group B (natural cycle, n=887): natural ovulation was monitored; Group C (Hormonal Replacement Therapy cycle, n=1,764): endometrial preparation was performed with estradiol valerate. It should be noted that only cycles that successfully completed embryo transfer were included in this study; cycles cancelled due to poor follicular development, ovulation disorders, suboptimal endometrial thickness, or other reasons were excluded. Therefore, cycle cancellation rates were not calculated in this analysis.

### 2.3 Endometrial preparation protocol

#### 2.3.1 Group A (OIC)

On days 3–5 of the menstrual cycle, initiate oral letrozole (Imetrex, Zhejiang Hisun Pharmaceutical Co., Ltd.) at a dose of 2.5–5 mg/day for 5 consecutive days. From days 8–10 of the menstrual cycle, perform transvaginal ultrasound to monitor follicular development and endometrial status, while measuring serum levels of LH, E2, and P. When the dominant follicle diameter reaches 18–20 mm and endometrial thickness is≥7 mm, administer human chorionic gonadotropin (HCG, Zhuhai Livzon Pharmaceutical) at 5,000-10,000 IU to trigger ovulation. The day of trigger is designated as P + 0. Depending on the embryonic stage, transfer a cleavage-stage embryo on P + 3 or a blastocyst on P + 5.

#### 2.3.2 Group B (NC)

Vaginal ultrasound monitoring of follicular development and endometrial status is initiated on days 8–10 of the menstrual cycle, along with measurement of serum LH, E2, and P levels. When the dominant follicle reaches a diameter of 18–20 mm, daily monitoring continues until ovulation. The day of ovulation is designated as P + 0. Depending on the embryonic stage, cleavage-stage embryos are transferred on P + 3, or blastocysts are transferred on P + 5.

#### 2.3.3 Group C (HRT)

On days 3–5 of the menstrual cycle, initiate oral administration of estradiol valerate tablets (Progynova, Bayer AG, Germany) at a starting dose of 4–6 mg/day, which may be gradually increased to 8–10 mg/day based on endometrial growth response. Endometrial monitoring should commence after 10–12 days of treatment. When endometrial thickness reaches≥7 mm, intramuscular progesterone injections are initiated to promote endometrial transformation. The day of progesterone initiation is designated as P + 0. Fertilized blastocysts should be transferred on P + 3, or blastocysts should be transferred on P + 5.

### 2.4 Luteal support protocol

Group A: Luteal support was initiated after ovulation. Progesterone soft capsules (Qining, Zhejiang Xianju Pharmaceutical Co., Ltd.) 200 mg were administered orally twice daily, and/or progesterone injection (Zhejiang Xianju Pharmaceutical Co., Ltd.) 40 mg was administered intramuscularly once daily; Group B: The same luteal support as Group A was initiated after ovulation; Group C: On day P + 0, initiate the same luteal support as Group A. All patients received continuous luteal support medication after embryo transfer until serum HCG levels were measured on day 14 post-transfer. If clinical pregnancy was confirmed, the medication was continued until 8–10 weeks post-transfer.

### 2.5 Embryo evaluation and transplantation

Both embryo freezing and thawing processes employed vitrification technology. The scoring of cleavage-stage embryos followed the Istanbul Consensus (18), with Grade I and Grade II embryos defined as high-quality embryos. The blastocyst scoring was performed using the Gardner scoring system (19), with blastocysts graded as 3BB or higher defined as high-quality embryos. All patients were selected for transfer based on age, embryo quality, and prior transfer history, with 1–2 high-quality embryos chosen for implantation.

### 2.6 Observation metrics and definitions

#### 2.6.1 Primary observation index

Clinical pregnancy rate: Vaginal ultrasound examination 28–35 days post-transplantation reveals an intrauterine gestational sac, embryonic pole, and primitive cardiac tube pulsations.

Clinical pregnancy rate = (Number of clinically confirmed pregnancy cycles/Total number of transplant cycles)×100%.

Live birth rate: The proportion of cycles resulting in live births.

Live birth rate = (Number of live birth cycles/Total number of transplantation cycles)×100%.

#### 2.6.2 Secondary outcome indicators

Durative pregnancy rate: A pregnancy that persists for more than 12 weeks after transplantation.

Early miscarriage rate: Clinical pregnancy termination before 12 weeks of gestation.

Ectopic pregnancy rate: Implantation of the gestational sac outside the uterine cavity.

Hormone levels: Serum E2 and P levels on the endometrial transformation day (HCG triggering day/ovulation day for Groups A and B, and the day of progesterone injection initiation for Group C) and embryo transfer day.

Endometrial thickness (EMT): Basal endometrial thickness, endometrial transformation thickness (T1), and embryo transfer day thickness (T2). EMT is measured as the maximum thickness of the bilateral anterior and posterior walls in the mid-sagittal plane of the uterus.

Endometrial morphology: According to the Gonen classification criteria (20), it is categorized into Type A (typical triple line sign), Type B (moderate echogenicity with blurred uterine cavity lines), and Type C (uniform high echogenicity with absent uterine cavity lines). The morphological classification on the endometrial transformation day and embryo transfer day is recorded.

Endometrial Compaction (EC): Calculate the rate of change in endometrial thickness from day P + 0 (T1) to embryo transfer day (T2). EC= [−(T2−T1) ÷T1] × 100%. According to the criteria in reference (21), an EC≥5% is defined as positive endometrial compaction.

### 2.7 Statistical analysis

All data analyses were performed using SPSS 27.0 statistical software.

Data Presentation and Grouping: Measurement data, after normality testing, were expressed as mean ± standard deviation ( $\bar{\text{x}}$ ± s). Count data were presented as case counts (percentage) [n (%)]. Patients were divided into three groups based on the endometrial preparation protocol: Group A (OIC), Group B (NC), and Group C (HRT). For further analysis, subgroups were defined according to the embryo transfer date (P + 3 or P + 5).

Inter-group comparisons: Comparative analysis of quantitative data among the three groups was performed using one-way ANOVA. If statistically significant differences were observed, *post-hoc* pairwise comparisons were conducted using the Bonferroni method. Comparative analysis of categorical data among the three groups was performed using the chi-square test or Fisher’s exact probability method.

Multivariate analysis: A binary logistic regression analysis was conducted to evaluate the independent impact of endometrial preparation protocols on clinical pregnancy and live birth outcomes. Based on recent clinical studies, age, BMI, AMH, total number of transferred embryos, and number of high-quality transferred embryos were included in the model as potential confounding factors for adjustment. Group C (Pregene replacement cycle) was used as the reference group to calculate the adjusted odds ratio (aOR) and its 95% confidence interval (CI).

**Figure 1:**
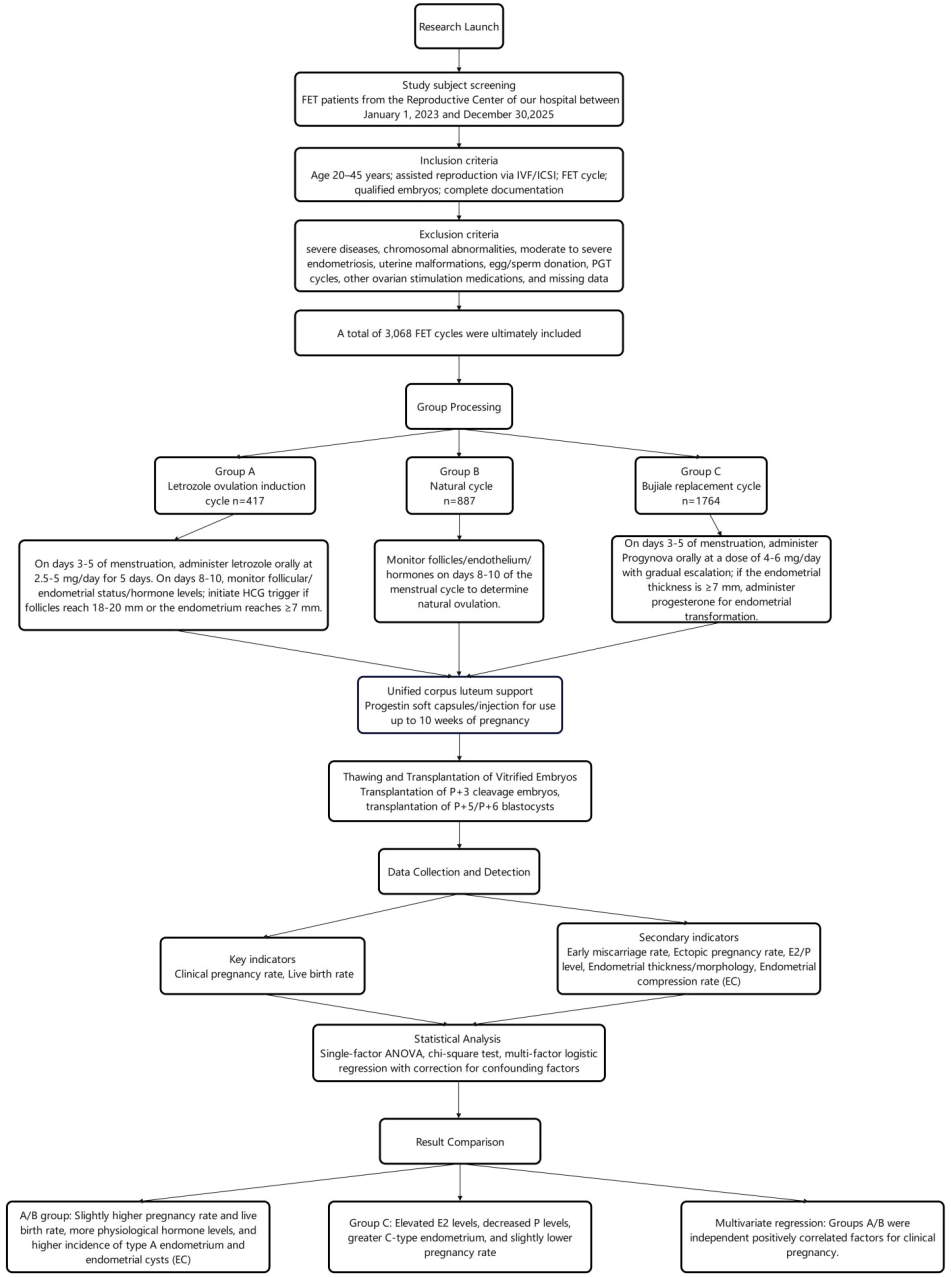
Flowchart of this study.

Statistical significance: All statistical analyses considered a P-value <0.05 as statistically significant (**Figure 1**).

## 3 Results

### 3.1 Patient baseline characteristics

A total of 3,068 FET cycles met the study criteria and were included in the analysis. Among these, Group A (OIC) comprised 417 cases (13.59%), Group B (NC) included 887 cases (28.91%), and Group C (HRT) consisted of 1,764 cases (57.5%). As shown in **Table 1**, there were no statistically significant differences among the three groups regarding baseline characteristics such as age, type of infertility, duration of infertility, infertility factors, BMI, baseline endocrine levels (FSH, LH, E2, P), AMH, baseline endometrial thickness, total number of transferred embryos, or number of high-quality transferred embryos (P> 0.05), indicating that the three groups were well-balanced and comparable.

**Table 1:** Comparison of baseline characteristics among the three patient groups.

Index		Group A	Group B	Group C	P
Age (y)		33.36 ± 4.72	33.38 ± 5.14	33.29 ± 5.19	0.905
BMI (kg/m²)		24.41 ± 4.51	23.99 ± 3.91	24.35 ± 4.20	0.075
Type of infertility	Primary infertility (%)	43.2% (180/417)	42.2% (374/887)	44.2% (780/1764)	0.597
	secondary infertility (%)	56.8% (237/417)	57.8% (513/887)	55.8% (984/1764)	
Duration of infertility (y)		3.36± 2.53	3.40 ± 2.60	3.46 ± 2.76	0.717
AMH (ng/ml)		3.35 ± 3.18	3.40 ± 3.22	3.36 ± 3.15	0.965
Base FSH (mIU/ml)		6.65 ± 4.12	7.14 ± 4.01	6.81 ± 4.03	0.063
Base LH (mIU/ml)		5.88 ± 4.01	5.49 ± 3.51	5.80 ± 5.07	0.194
Base E2 (pg/ml)		41.63 ± 34.00	45.02 ± 35.51	43.97 ± 34.15	0.255
Base P (ng/ml)		0.58 ± 0.80	0.57 ± 0.57	0.60 ± 0.74	0.577
Transplantation intimal thickness (mm)		9.99 ± 1.98	9.94 ± 2.04	10.03 ± 2.01	0.569
Number of transferred embryos		1.67 ± 0.47	1.66 ± 0.48	1.66 ± 0.48	0.712
Number of high-quality embryos transplanted		0.46 ± 0.52	0.47 ± 0.54	0.50 ± 0.54	0.139
Number of previous transplantations		0.72 ± 1.12	0.70 ± 1.15	0.79 ± 1.20	0.328

### 3.2 Periodic characteristics, hormone levels, and endometrial parameters

The cyclical characteristics and hormone level comparisons among the three patient groups are presented in **Table 2**.

**Table 2:** Comparison of cycle characteristics and hormone levels among the three patient groups.

Index		Group A	Group B	Group C	P
Endometrial Transformation Day	E2 (pg/ml)	285.45 ± 150.32*	278.65 ± 145.78*	395.67 ± 210.45	<0.001
	P (ng/ml)	1.25 ± 0.85*	1.32 ± 0.88*	0.45 ± 0.32	<0.001
	EMT (mm)	9.1 ± 1.6*	9.0 ± 1.5*	9.8 ± 1.8	<0.001
	Type A endocardium (%)	75.5% * (315/417)	77.1%* (685/887)	72.1% (1272/1764)	0.014
	Type B endocardium (%)	22.8% (95/417)	21.5% (191/887)	20.1% (354/1764)	0.397
	Type C endocardium (%)	1.7% (7/417)	1.4% (11/887)	7.8% (138/1764)	<0.001
Embryo Transfer Day	E2 (pg/ml)	215.38 ± 120.15*	208.45 ± 115.32*	350.12 ± 185.64	<0.001
	P (ng/ml)	10.58 ± 6.12	10.62 ± 6.08	10.54 ± 6.27	0.95
	EMT (mm)	8.8 ± 1.5*	8.7 ± 1.4*	9.5 ± 1.7	<0.001
	Type A endocardium (%)	1.2% (5/417)	1.4% (12/887)	0.7% (12/1764)	0.203
	Type B endocardium (%)	28.5% (119/417)	27.8% (247/887)	29.0% (512/1764)	0.818
	Type C endocardium (%)	70.3% (293/417)	70.8% (628/887)	70.3% (1240/1764)	0.96
Duration of estrogen administration (d)		12.1 ± 3.5	12.3 ± 3.6	11.9 ± 3.3	0.122
Endometrial compression rate (≥5%)		35.0% (146/417)*	34.0% (302/887)*	28.6%(504/1764)	<0.001

* indicates a statistically significant difference compared to Group C (P <0.05).

Regarding hormone levels, the serum E2 levels in Groups A and B on endometrial transformation day were significantly lower than those in Group C (395.67 ± 210.45 pg/ml) (P <0.001), measuring 285.45 ± 150.32 pg/ml and 278.65 ± 145.78 pg/ml, respectively. The serum P levels in Groups A and B (1.25 ± 0.85 ng/ml and 1.32 ± 0.88 ng/ml) were significantly higher than those in Group C (0.45 ± 0.32 ng/ml) (P <0.001), while no significant difference was observed between Groups A and B (P> 0.05). On embryo transfer day, the E2 levels in Groups A and B (215.38 ± 120.15 pg/ml and 208.45 ± 115.32 pg/ml) remained significantly lower than those in Group C (350.12 ± 185.64 pg/ml) (P <0.001), whereas no significant difference was noted in P levels among the three groups (P> 0.05).

Regarding endometrial parameters, the endometrial thickness (EMT) in Groups A and B on the endometrial transformation day was (9.1 ± 1.6) mm and (9.0 ± 1.5) mm, respectively, both thinner than that in Group C (9.8 ± 1.8) mm (P <0.001). In terms of endometrial morphology, the proportions of type A endometrium in Groups A and B (75.5% and 77.1%) were significantly higher than that in Group C (72.1%) (P = 0.014); although the proportion of type B endometrium in Group C (20.1%) was slightly lower than that in Group A (22.8%) and Group B (21.5%), the difference was not statistically significant (P> 0.05); whereas the proportion of type C endometrium in Group C (7.8%) was significantly higher than that in Group A (1.7%) and Group B (1.4%) (P <0.001). On the embryo transfer day, the EMT in Groups A and B remained thinner than that in Group C (P <0.001), and at this stage, the endometrial morphology in all three groups was predominantly type C, with no significant intergroup differences (P> 0.05). The duration of estrogen administration did not show statistically significant differences among Groups A (12.1 ± 3.5 days), B (12.3 ± 3.6 days), and C (11.9 ± 3.3 days) (P> 0.05).

Regarding endometrial compression, the proportion of endometrial compression (EC ≥ 5%) was 35.0% (146/417) in Group A, 34.0% (302/887) in Group B, and 28.6% (504/1764) in Group C. The EC incidence rates in both Groups A and B were significantly higher than that in Group C (P <0.001), whereas no significant difference was observed between Groups A and B (P> 0.05).

### 3.3 Comparison of pregnancy outcomes

**Figure 2:**
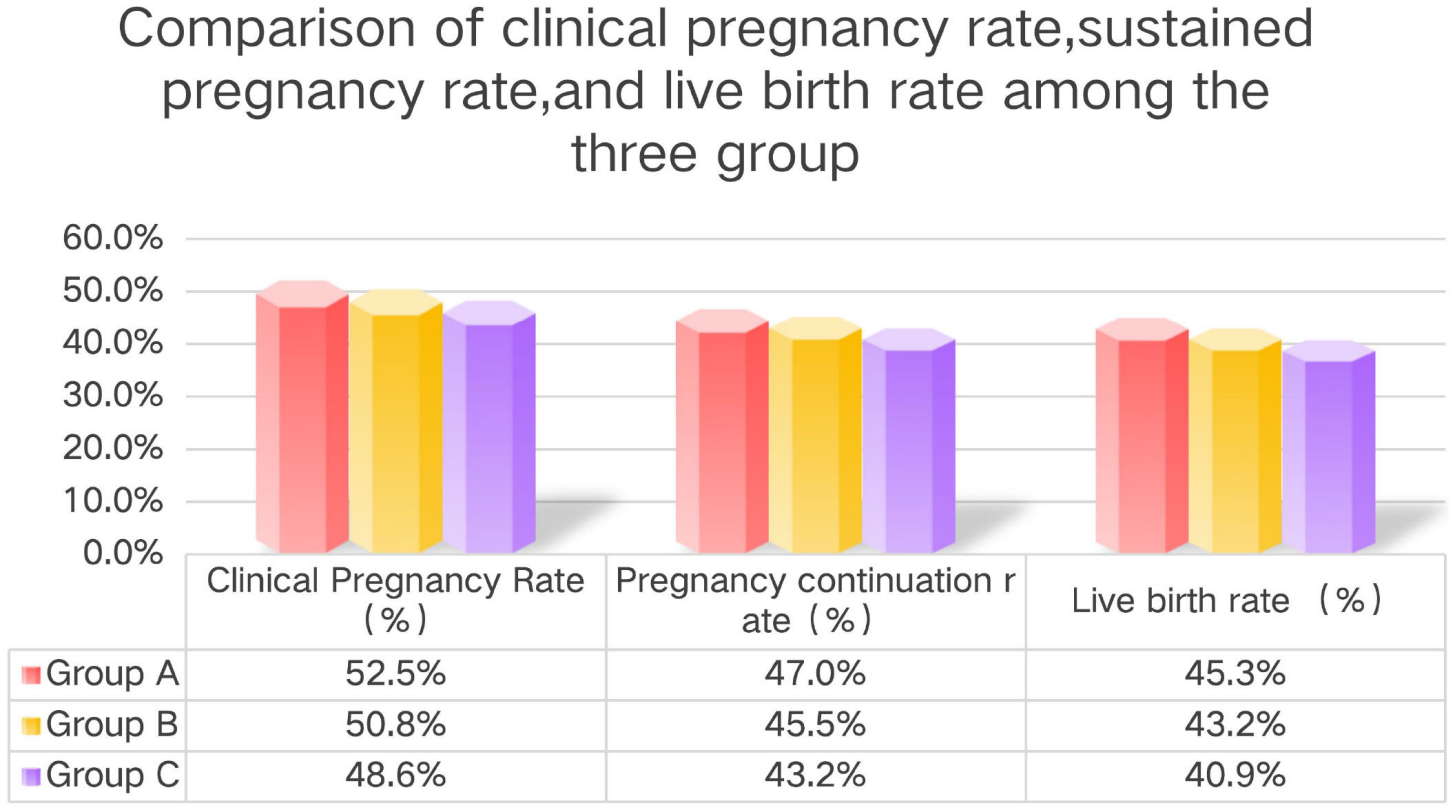
Comparison of clinical pregnancy rate, sustained pregnancy rate, and live birth rate among the three groups.

The comparison of pregnancy outcomes among the three patient groups is presented in **Table 3** and **Figure 2**.

**Table 3:** Comparison of pregnancy outcomes among the three patient groups.

Index	Group A	Group B	Group C	P
Clinical Pregnancy Rate (%)	52.5% (219/417)	50.8% (451/887)	48.6% (858/1764)	0.275
Pregnancy continuation rate (%)	47.0% (196/417)	45.5% (404/887)	43.2% (762/1764)	0.266
Live birth rate (%)	45.3% (189/417)	43.2% (383/887)	40.9% (722/1764)	0.204
Early abortion rate (%)	10.5% (23/219)	10.4% (47/451)	11.2% (96/858)	0.898
Ectopic pregnancy rate (%)	1.4% (6/417)	1.1% (10/887)	1.6% (29/1764)	0.579

* indicates a statistically significant difference compared to Group C (P <0.05).

Regarding the primary outcomes, the clinical pregnancy rates in Group A (52.5%,219/417) and Group B (50.8%,451/887) were higher than those in Group C (48.6%,858/1764), but the difference was not statistically significant (P> 0.05). The live birth rates in Group A (45.3%,189/417) and Group B (43.2%,383/887) were also higher than those in Group C (40.9%,722/1764), with similarly non-statistically significant differences (P> 0.05). For secondary outcomes, the continued pregnancy rate was higher in Groups A (47.0%,196/417) and B (45.5%,404/887) than in Group C (43.2%,762/1764), but the difference was not statistically significant (P> 0.05). The early miscarriage rate was lower in Groups A (10.5%,23/219) and B (10.4%,47/451) than in Group C (11.2%,96/858), with no statistically significant difference either (P> 0.05). There were no significant differences in the ectopic pregnancy rates among the three groups (P> 0.05).

### 3.4 Subgroup analysis

Subgroup analysis was performed based on the type of transplanted embryo (blastocyst vs blastula), with results presented in **Table 4**.

**Table 4:** Subgroup analysis at different embryonic stages.

Subunit	Group A	Group B	Group C	P
P+3 cycle	n=202	n=457	n=905	
Clinical Pregnancy Rate (%)	49% (99/202)	49.7% (227/457)	47.7% (432/905)	0.785
P+5 cycle	n=202	n=398	n=809	
Clinical Pregnancy Rate (%)	55.9% (113/202)	52% (207/398)	49.6% (401/809)	0.250
P+6 cycle	n=13	n=32	n=50	
Clinical Pregnancy Rate (%)	53.8% (7/13)	53.1% (17/32)	50% (25/50)	0.948

In the cleavage-stage embryo transfer (P + 3) subgroup, the clinical pregnancy rates in Group A (3A) and Group B (3B) were 49% and 49.7%, respectively, both higher than those in Group C (3C,47.7%), though the difference was not statistically significant (P = 0.785). In the blastocyst-stage embryo transfer (P + 5) subgroup, the clinical pregnancy rates in Group A (5A) and Group B (5B) were 55.9% and 52%, respectively, also higher than those in Group C (5C,49.6%), but the difference was not statistically significant (P = 0.250). In the blastocyst-stage embryo transfer (P + 6) subgroup, the clinical pregnancy rates in Group A (6A) and Group B (6B) were 53.8% and 53.1%, respectively, similarly higher than those in Group C (6C,50%), but the difference was not statistically significant (P = 0.948), and given the small sample size, the results should be interpreted with caution.

### 3.5 The relationship between endometrial compression and pregnancy outcomes

Further analysis of the impact of endometrial compression on pregnancy outcomes is presented in **Table 5**.

**Table 5:** Relationship between endometrial compression and clinical pregnancy rate.

Group	Endometrial compression (EC ≥ 5%)	Endometrial non-compression (EC <5%)	P
Group A (letrozole-induced ovulation cycle, n=417)	56.8% (83/146)	50.2% (136/271)	0.194
Group B (natural cycle, n=887)	55.6% (168/302)	48.4% (283/585)	0.041
Group C (Hormonal Replacement Therapy cycle, n=1764)	52.2% (263/504)	47.2% (595/1260)	0.060
合计	54.0% (514/952)	47.9% (1014/2116)	0.002

In all cycles, the clinical pregnancy rate was 54.0% (514/952) in patients with endometrial compression (EC ≥ 5%), significantly higher than that in patients without endometrial compression (47.9% [1014/2116]) (P <0.05). Within all three groups, the clinical pregnancy rate was higher in patients with endometrial compression compared to those without compression, with the difference being particularly pronounced in Group B.

### 3.6 Multivariate logistic regression analysis of factors influencing clinical pregnancy

Using clinical pregnancy as the dependent variable, the endometrial preparation protocol (with Group C as the reference), age, BMI, AMH levels, total number of transferred embryos, and number of high-quality transferred embryos were included as independent variables in a binary logistic regression model. The results are presented in **Table 6**. After adjusting for potential confounding factors, Group A (OIC) was an independent positive predictor of clinical pregnancy (aOR = 1.254; 95% CI 1.032–1.525; P = 0.023). Group B (NC) was also an independent positive predictor of clinical pregnancy (aOR = 1.312; 95% CI 1.045–1.648; P = 0.019). Additionally, higher AMH levels (aOR = 1.058; 95% CI 1.020–1.098; P = 0.002) and a greater number of high-quality transferred embryos (aOR = 1.345; 95% CI 1.120–1.615; P = 0.001) were favorable factors for clinical pregnancy, whereas advanced age (aOR = 0.955; 95% CI 0.930–0.981; P = 0.001) was a risk factor.

**Table 6:** Binary logistic regression analysis of factors affecting clinical pregnancy.

Variable		aOR	95% confidence interval	P
Age		0.955	[0.930, 0.981]	0.001
BMI		0.998	[0.971, 1.026]	0.892
AMH		1.058	[1.020, 1.098]	0.002
Total number of transplanted embryos		1.085	[0.892, 1.320]	0.412
Number of high-quality embryos transplanted		1.345	[1.120, 1.615]	0.001
Endometrial Preparation Protocol	Group C	1000 (Reference)		
	Group A	1.254	[1.032, 1.525]	0.023
	Group B	1.312	[1.045, 1.648]	0.019

## 4 Discussion

This study conducted a systematic comparison of the clinical efficacy of letrozole ovulation induction cycles, natural cycles, and Hormonal Replacement Therapy cycle in FET through a single-center, large-sample retrospective cohort analysis. The main findings include: (1) The clinical pregnancy rate and live birth rate were numerically higher but non-significant in letrozole cycles and natural cycles compared to Hormonal Replacement Therapy cycle;(2) The hormonal environment in letrozole cycles and natural cycles was closer to physiological conditions, characterized by lower endometrial transformation day E2 levels and higher progesterone (P) levels; (3) The proportion of type A endometrium and the incidence of endometrial compaction were higher in letrozole cycles and natural cycles; (4) Multivariate analysis indicated that both letrozole cycles and natural cycles were independent positive predictors of clinical pregnancy compared to Hormonal Replacement Therapy cycle.

The results of this study demonstrate that the E2 levels on the endometrial transformation day were significantly lower in the letrozole cycle and the natural cycle compared to Hormonal Replacement Therapy cycle, while the P levels were significantly higher. This finding suggests that both regimens create a hormonal environment more closely resembling a natural ovulatory cycle. In Hormonal Replacement Therapy cycle, higher doses of exogenous estrogen are often required to achieve adequate endometrial thickness, resulting in supra-physiological levels of E2 exposure (22). Existing studies have demonstrated that excessively high E2 levels may adversely affect endometrial receptivity, including altering the expression of genes associated with the implantation window (e.g., LIF, HOXA10), impairing endometrial blood flow and angiogenesis, and disrupting immune cell function, thereby leading to an imbalance in the embryo-endometrium dialogue (23, 24). The higher E2 levels observed in the control group of this study may partially explain its relatively lower pregnancy rate (25). On the other hand, the functional corpus luteum formed after ovulation from the own follicle during letrozole cycles and natural cycles secretes endogenous progesterone. This not only provides corpus luteum support but may also release other bioactive substances such as relaxin and vascular endothelial growth factor, collectively optimizing endometrial receptivity (26, 27). Although there were no significant differences in serum P levels among the three groups on the embryo transfer day, local tissue P concentrations and receptor function may vary. The Hormonal Replacement Therapy cycle is entirely dependent on exogenous P, posing a potential risk of “progesterone resistance,” i.e., reduced endometrial responsiveness to progesterone (28–30).

In terms of endometrial morphology, although the average EMT during letrozole treatment cycles and natural cycles was slightly thinner than that during the combined oral contraceptive cycle, the proportion of type A endometrium was significantly higher. Type A endometrium (characterized by the classic three-line pattern) is generally considered the ideal morphology from the late proliferative phase to the early secretory phase, associated with higher receptivity and pregnancy rates (31). This suggests that the “quality” of the endometrium may be more critical than mere “thickness.” As an aromatase inhibitor, letrozole suppresses estrogen synthesis while transiently elevating androgen levels in the ovaries. Androgens can promote proliferation and differentiation of endometrial glands and stroma through their receptors, potentially contributing to the formation of a superior endometrial morphology and ultrastructure (32, 33). Endometrial compression (EC) has emerged as a prominent receptivity indicator in recent years, referring to the moderate reduction in endometrial thickness between endometrial transformation and the transplantation date. It is considered indicative of the endometrium’s optimal response to progesterone and adequate glandular secretory transformation (34, 35). This study found that the incidence of endometrial cancer (EC) during letrozole treatment cycles and natural cycles was significantly higher than during the HRT cycle, and patients who developed EC exhibited a higher clinical pregnancy rate. These findings further support that letrozole and natural cycles can induce a more physiological progesterone response, promoting adequate endometrial transition to the secretory phase.

Subgroup analysis in this study revealed that during blastocyst transfer cycles, the clinical pregnancy rates were numerically higher in the letrozole cycle and natural cycle compared to Hormonal Replacement Therapy cycle. In cleavage-stage transfer cycles, the differences among the three groups were smaller; however, none of the subgroup differences reached statistical significance. Nevertheless, this phenomenon warrants attention. Possible explanations include: (1) Blastocysts require higher endometrial synchronicity, necessitating more precise matching of the “implantation window” (36, 37); (2) The physiological hormonal environment provided by letrozole treatment and the natural menstrual cycle may be more conducive to blastocyst implantation; (3) Blastocyst transfer during the cleavage stage requires shorter *in vitro* embryo culture times and exhibits relatively lower dependence on endometrial receptivity. This finding holds clinical implications: for patients scheduled for blastocyst transfer, letrozole-treated cycles or the natural cycle may demonstrate potential advantages, though further validation through larger sample sizes or prospective studies is warranted.

Based on the findings of this study, letrozole ovulation induction cycles and natural cycles achieved similar clinical pregnancy rates and live birth rates in FET, with both values being numerically superior to those observed in hormone replacement therapy cycles, but non-significant. Similar pregnancy outcomes were achieved with both approaches. The choice between the two should be tailored to the patient’s specific circumstances: natural cycles require no exogenous hormones, closely resemble physiological conditions, and are cost-effective; however, they necessitate frequent ovulation monitoring and have a relatively higher cycle cancellation rate (e.g., due to ovulatory disorders or premature ovulation), making precise scheduling of embryo transfer dates more challenging (38, 39). The letrozole cycle induces follicular development through pharmacological intervention, enabling partial regulation of ovulation timing with superior cycle controllability compared to the natural cycle, while preserving the physiological advantages of endogenous hormone production. For patients with irregular menstruation or poor follicular development, the letrozole cycle may demonstrate greater advantages over the natural cycle (15). Although the Hormonal Replacement Therapy cycle exhibits a slightly lower pregnancy rate, it remains an effective alternative option for patients with ovulation disorders, poor follicular development, thin endometrium, or those requiring precise scheduling of embryo transfer dates (40). For patients with recurrent implantation failure, letrozole or natural cycle may be considered to improve endometrial receptivity (41, 42).

### 4.1 Advantages and limitations

The advantages of this study are primarily reflected in the following aspects: First, the sample size is substantial, encompassing a total of 3,068 FET cycles, making it one of the largest studies currently comparing three endometrial preparation protocols. Second, the study design is comprehensive, comparing all three protocols while systematically analyzing receptivity indicators such as hormone levels, endometrial parameters, and endometrial compression. Additionally, through in-depth subgroup analyses conducted for different embryonic stages, the study provides a reference basis for clinical individualized selection. Finally, multivariate regression analysis was employed to correct for potential confounding factors, effectively controlling for confounding bias and enhancing the reliability of the conclusions.

However, this study also has certain limitations. First, the study employed a retrospective design, which inherently introduces selection bias, although we made every effort to control confounding factors during analysis. As a retrospective study, residual confounding cannot be completely excluded despite multivariate adjustment, and causal relationships should be interpreted with caution. Second, as a single-center study, the generalizability of our findings to other populations with different demographic characteristics or clinical practices may be limited. Third, endometrial receptivity was evaluated only by ultrasound morphology and hormone levels; molecular markers such as ERA or endometrial gene expression profiling were not performed, limiting our ability to assess receptivity at the tissue level. Fourth, serum progesterone (P) levels may differ from local endometrial P concentrations and receptor function; serological markers cannot fully reflect the true tissue-level status. Fifth, no health economics evaluation was conducted, leaving a comparison of cost-effectiveness among different treatment regimens unakukan. Sixth, although trends in differences among the three groups for the primary outcome indicators (clinical pregnancy rate and live birth rate) were observed, these differences were not statistically significant. Therefore, the conclusions of this study should be regarded as exploratory findings and require further validation through prospective studies.

## 5 Conclusion

This study, through a single-center, large-sample retrospective cohort analysis, yielded the following conclusions: During frozen embryo transfer (FET) cycles, the clinical pregnancy and live birth rates of letrozole ovulation induction cycles and natural cycles were numerically higher than those of Hormonal Replacement Therapy cycle, though the differences were not statistically significant. Both regimens provided a hormonal environment closer to physiological conditions (e.g., lower estradiol levels and higher endogenous progesterone levels) and superior endometrial receptivity indicators (e.g., higher proportions of type A endometrium and lower rates of endometrial compression). Subgroup analyses suggested more pronounced advantages of letrozole cycles and natural cycles during blastocyst transfer cycles, although these differences also lacked statistical significance. The study identified an endometrial compression degree of 5% or higher as a favorable predictor of clinical pregnancy and a valuable adjunct tool for assessing endometrial receptivity. Based on these findings, letrozole ovulation induction cycles and natural cycles are recommended as preferred endometrial preparation regimens for patients with ovulatory function. However, clinicians should fully inform patients that current data do not demonstrate statistically significant differences and emphasize the need for individualized decision-making considering factors such as ovulatory function, endometrial response, and cycle regularity.

Future research should include more prospective, multicenter, randomized controlled studies, combined with molecular biological markers (such as ERA testing and endometrial gene expression profiling) to further validate the trend findings of this study and elucidate the molecular mechanisms underlying the effects of different treatment regimens on endometrial receptivity. Additionally, health economics evaluations should be strengthened to provide more comprehensive evidence-based support for clinical decision-making.

## Data Availability

The raw data supporting the conclusions of this article will be made available by the authors, without undue reservation.
